# Acetylation of C-terminal lysines modulates protein turnover and stability of Connexin-32

**DOI:** 10.1186/s12860-018-0173-0

**Published:** 2018-09-29

**Authors:** Sarah R. Alaei, Charles K. Abrams, J. Chloë Bulinski, Elliot L. Hertzberg, Mona M. Freidin

**Affiliations:** 1 Department of Molecular Genetics & Microbiology, Stony Brook University, Stony Brook, NY 11794 USA; 2 Department of Neurology & Rehabilitation, University of Illinois at Chicago, Chicago, IL 60612 USA; 3 Department of Cell & Molecular Biology, Columbia University, New York, NY 10032 USA

**Keywords:** Gap junctions, Acetylation, Ubiquitination, Cell-cell communication, Connexin

## Abstract

**Background:**

The gap junction protein, Connexin32 (Cx32), is expressed in various tissues including liver, exocrine pancreas, gastrointestinal epithelium, and the glia of the central and peripheral nervous system. Gap junction-mediated cell-cell communication and channel-independent processes of Cx32 contribute to the regulation of physiological and cellular activities such as glial differentiation, survival, and proliferation; maintenance of the hepatic epithelium; and axonal myelination. Mutations in Cx32 cause X-linked Charcot–Marie–Tooth disease (CMT1X), an inherited peripheral neuropathy. Several CMT1X causing mutations are found in the cytoplasmic domains of Cx32, a region implicated in the regulation of gap junction assembly, turnover and function. Here we investigate the roles of acetylation and ubiquitination in the C-terminus on Cx32 protein function. Cx32 protein turnover, ubiquitination, and response to deacetylase inhibitors were determined for wild-type and C-terminus lysine mutants using transiently transfected Neuro2A (N2a) cells.

**Results:**

We report here that Cx32 is acetylated in transfected N2a cells and that inhibition of the histone deacetylase, HDAC6, results in an accumulation of Cx32. We identified five lysine acetylation targets in the C-terminus. Mutational analysis demonstrates that these lysines are involved in the regulation of Cx32 ubiquitination and turnover. While these lysines are not required for functional Cx32 mediated cell-cell communication, BrdU incorporation studies demonstrate that their relative acetylation state plays a channel-independent role in Cx32-mediated control of cell proliferation.

**Conclusion:**

Taken together these results highlight the role of post translational modifications and lysines in the C-terminal tail of Cx32 in the fine-tuning of Cx32 protein stability and channel-independent functions.

**Electronic supplementary material:**

The online version of this article (10.1186/s12860-018-0173-0) contains supplementary material, which is available to authorized users.

## Background

Connexins are a family of 21 homologous integral membrane proteins that form cell-cell channels, known as gap junctions (GJ) [1–3]. GJ provide a low resistance pathway for the diffusion of small molecules and ions between coupled cells [4]. Recent data also suggest connexin involvement in channel-independent processes including cell growth, autophagosome formation, cell adhesion, cell motility and cell migration [5–10]. The C-termini of different connexins vary substantially in length and in their capacity to mediate interactions with the cytoskeleton [11–13], and junctional complexes [12, 14]. The C-terminal sequences of connexins have also been implicated in voltage (reviewed in [15]), pH and chemical [16–18], gating of different GJ channels. C-terminal truncation of GJA1 (Connexin43; Cx43) does not alter the ability to form functional gap junctions, but does alter trafficking to the plasma membrane and gap junction plaque formation to indirectly reduces overall GJ-mediated cell-cell communication [19–21]. Cytoplasmic domains in several connexins, including Cx43 and GJB1 (Connexin32; Cx32), have also been implicated in GJ-independent processes, such as regulation of cell growth and gene expression [22–24].

The cytoplasmic domains of some connexins are subject to post translational modifications such as phosphorylation, ubiquitination, and acetylation, though relatively little is known about how these modifications impact function. To date, most investigations of connexin post translational modifications have focused on Cx43. Phosphorylation modulates Cx43-mediated GJ communication through the modulation of channel closure [25], accretion in GJ plaques [25, 26], removal from the plasma membrane, and subsequent protein turnover [27, 28]. Ubiquitinated Cx43 is targeted for degradation by the proteasomal [29, 30], endo-lysosomal [31], and autophagosomal [32] pathways, depending on its subcellular localization, specific ubiquitin moieties and modification sites. Acetylation of Cx43 has also been identified as a negative regulator of plasma membrane localization [33], although it is unclear if it influences Cx43 insertion or degradation. Despite having been identified as a phosphoprotein over 20 years ago [34], considerably less is known about the abundance and functions of any post-translational modifications of Cx32.

Cx32 is widely expressed in various tissues including liver, exocrine pancreas, gastrointestinal epithelium, and the glia of the central and peripheral nervous system. Mutations in Cx32 cause X-linked Charcot–Marie–Tooth disease (CMT1X), an inherited peripheral neuropathy [35, 36]. CMT1X associated mutations result in diverse alterations in Cx32 expression and function, including defects in trafficking and GJ formation and gating [35, 37–39]. More than 300 CMT1X mutations have been identified covering most of the Cx32 gene (http://www.molgen.ua.ac.be/CMTMutations). CMT1X mutations in the transmembrane and cytoplasmic loop regions prevent the assembly of GJ in the plasma membrane CMT1X [40–42]. Mutations in the C-terminus of Cx32 result in functional alterations but do not prevent GJ assembly [38].

The C-terminus of Cx32 has also been implicated in processes such as Ca^2+^ regulated gap junctional gating [43] and the modulation of cellular proliferation [44]. Post-translational modifications have been identified in the C-terminus of Cx32, but their physiological significance is poorly understood [45, 46]. In this study, we focus on the role of post translational modifications of C-terminal lysine residues in the modulation of Cx32 protein stability and function. We report here that Cx32 is acetylated and have identified five lysines in the C-terminus as likely acetylation sites. Further, we demonstrate that these lysines are involved in the regulation of Cx32 protein stability, ubiquitination, and turnover. Finally, while the acetylation state of these lysines are not directly involved in modulating Cx32 GJ communication, we show their relative acetylation state plays a role in Cx32-mediated control of channel-independent cellular processes such as cell proliferation.

## Methods

### Cell lines

**Fig. 1:**
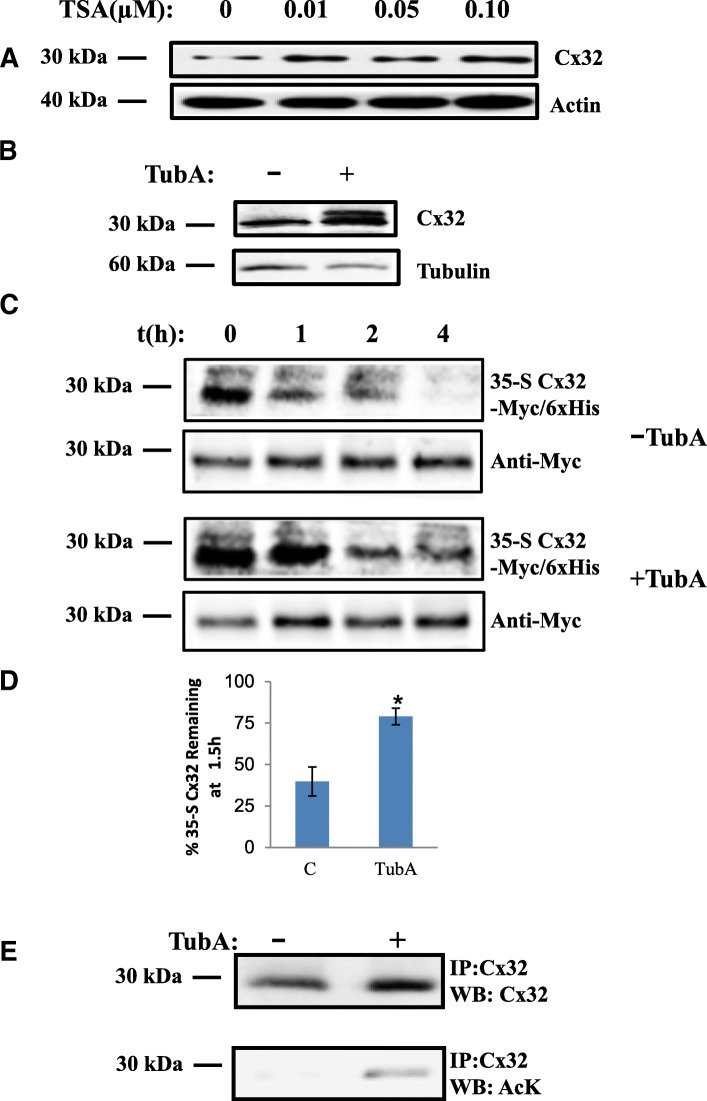
HDAC6 inhibition reduces turnover and increases acetylation of Cx32. **a** TSA and (**b**) TubA increase expression level of Cx32. Western blots of N2a cell lysates treated with the TSA (concentrations as indicated) (**a**) or 20 μM TubA for 6 h (**b**) were probed with anti-Cx32, actin (**a**) or tubulin (**b**) was used as a loading control. **c** TubA treatment decreases turnover of Cx32 (bottom panel) compared to untreated samples (top panel). Protein turnover of 35-S-Cx32 was determined by phosphorimaging at 0, 1, 2, 4 h post-chase (shown). Blots were subsequently probed with anti-Myc as a loading control. **d** Relative levels of 35-S-Cx32 remaining after 1.5 h post-chase (in subsequent experiments) was quantified by densitometry of phosphorimages, normalized to Myc, and used to calculate half-life, as described (see Methods). (*N* = 4 independent experiments; student’s T-test* *p* < 0.05 as compared to DMSO control). **e** TubA increases expression levels of total Cx32 and acetylated Cx32. Cx32 immunoprecipitates of N2a cell lysates expressing Cx32-Myc/6xHis treated with TubA or vehicle alone were processed for Western blots analysis and probed with Cx32 and AcK antibodies

Cell lines were obtained from American Type Culture Collection (ATCC, Manassas, VA). Cultures were maintained in DMEM supplemented with 10% FBS at 37 °C and 5% CO2, at 100% humidity. Unless otherwise noted, all experiments were performed using connexin-deficient Neuro-2a (N2a) cells (ATCC #CCL-131). N2a cells were used for subsequent experiments to maintain consistency between electrophysiological, microscopic, and biochemical analyses. HeLa cells (ATCC #CCL-2) were used in preliminary experiments (Fig. 1a, b).

### Plasmids

Rat Cx32 cDNA subcloned into pIRES2EGFP**.** To generate Myc/His-tagged constructs, rat Cx32 cDNA was cloned into BamHI/ApaI sites of pcDNA3.1/Myc-His A. The K➔R and K➔Q mutations were generated using Genetailor site-directed mutagenesis kit, as per manufacturer’s recommendations (Invitrogen/Life Technologies) and subcloned into relevant vectors. pIRES2EGFP-Cx32 was used for imaging, BrdU incorporation, and electrophysiology experiments. pcDNA3.1-Cx32-Myc/His was used in all other experiments to facilitate isolation of Cx32 using the Myc and His epitope tags.

### Western blotting

Total protein from N2a cells was harvested in PBS supplemented with 1% Triton-X 100. Samples were denatured 30 min at room temperature following addition of standard Laemmli SDS-PAGE loading buffer to limit aggregate formation [47, 48]. Proteins were separated on 10% polyacrylamide denaturing gels and transferred to nitrocellulose membranes. Blocking and antibody dilution was carried out in PBS containing 2% BSA and 0.05% Tween.

Western blots were probed with mouse anti-Cx32 clone 7c6.7c (Elliot Hertzberg) 1:1000, rabbit anti-Myc tag (AbCam) 1:2000, rabbit anti-acetylated proteins (Ack, Cell Signaling Technologies) 1:1000, mouse anti-ubiquitinated proteins clone P4G7 (Covance)1:200, and mouse anti-β-tubulin clone 3F3-G2 at 1:2000. Goat anti-rabbit IRdye800 and goat anti-mouse IRdye700 secondary antibodies (LiCor) were used for detection and digital images were obtained with the Odyssey imaging system (LiCor).

Western blots were also analyzed using GelQuant.NET software (http://biochemlabsolutions.com/GelQuantNET.html), normalizing to actin or β-tubulin signal, where appropriate. Fold change was calculated relative to untreated WT signals for each probe.

### Drugs and other chemicals

TubastatinA (TubA, Chemie Tek) and Trichostatin A (TSA, Sigma) were dissolved in DMSO (1 mM and 20 μM, respectively) prior to further dilution in medium. All other chemicals were obtained from Sigma Aldrich, unless otherwise indicated. Cells were treated with 20 μM TubA for 3 h, unless otherwise indicated.

### Transfections

N2a cells were transiently transfected using Lipofectamine LTX (Invitrogen/Life Technologies) according to the manufacturer’s instructions. Antibiotic-free media was used for transfection reactions and all subsequent culture steps. Transfected cells were re-seeded on to glass coverslips at 12–16 h post-transfection for electrophysiology and immunofluorescence experiments.

### Immunofluorescence and BrdU incorporation

Transfected cells were grown overnight after seeding on to glass coverslips, treated as described, and fixed 15-min at room temperature with Histochoice (Amresco). Coverslips were washed with PBS supplemented with 0.05% Tween. Blocking and antibody dilutions were carried out in PBS supplemented with 0.05% Tween and 2% BSA. Cx32 was probed with mouse anti-Cx32 clone 7C6.6 (1:1200). Signal was detected with Alexa-Fluor-conjugated secondary antibodies (Molecular Probes) and the coverslips were mounted using ProLong Gold plus DAPI (Molecular Probes) or Fluoromount (Invitrogen) with DAPI.

For proliferation studies, cultures were treated with BrdU (10 μM, Molecular Probes) overnight and fixed with Histochoice as described. Immunofluorescent detection of Cx32 preceded denaturation and immunolabeling for BrdU. Following immunolabeling for Cx32, samples were denatured 30 min with 2 N HCl in 0.1% Triton-X-100, neutralized with 0.2 M Borate buffer, pH 9.0 and washed with PBS. The samples were then processed for BrdU incorporation using monoclonal anti-BrdU (LabVision/ThermoFisher;1:150) immunofluorescence.

### Imaging

A Zeiss LSM 700 confocal microscope system was used to obtain Z-stacks at 63× magnification. All images were obtained with a pinhole size of 1 airy unit. Laser power, filter settings, beam splitter, and scan mode were the same for all images. Confocal imaging was used to obtain Z-stacks of 0.29um slices (63X). Images shown are maximum intensity projections of 5 slices that were most in focus at points of cell-cell contact. Integrated fluorescence values were obtained from background subtracted images using the Cell Scoring and Multi-Wavelength Cell Scoring applets in Metamorph 7 image analysis software (Molecular Devices). Thresholds were determined using negative control images and cellular integrated fluorescence constituted less than 10% of positively scored cells.

### Cell lysate preparation for immunoprecipitation and Co2+ purification

Cells were scraped into PBS and collected by low speed centrifugation. The cell pellets were lysed in PBS supplemented with 1% Triton-X 100 and protease inhibitors (LB). 20% SDS was added to a final concentration of 1.2% and incubated on ice 30 min to solubilize gap junctions [49]. For immunoprecipitation, sufficient LB was added to dilute SDS to a final concentration of 0.4%. The lysate was passed through a 26.5-gauge needle and centrifuged to remove debris. For His-tagged Cx32, Hispur-Co2+ coated agarose beads (Pierce) were added to the cleared supernatant and incubated as per manufacturer’s suggestions. His-tagged Cx32 was eluted with lysis buffer containing 300 mM imidazole, as per manufacturer’s suggestions. Where noted, samples were immunoprecipitated using an affinity purified rabbit polyclonal to Cx32 raised to C-terminal peptides (E. Hertzberg) with Protein G-agarose beads (Pierce/ThermoFisher) and eluted as per manufacturer’s suggestions.

### 35-S met/Cys pulse-chase assay

Cells were transfected 48 h prior to experiments. Each experimental sample consisted of confluent 10 cm plate of transfected cells. For +/− TubA experiments, cells were pretreated with 20 μM TubA or DMSO for 16 h prior to Met/Cys pulse. Cells were rinsed with 3 × 4 ml PBS and then starved in DMEM lacking serum, methionine, and cysteine for 1 h. The 35-S-Met/Cys pulse was carried out for 20 min (+/− TubA experiments) or five minutes (WT versus 5R or 5Q).

Radioactive pulse media containing 50 mCi/ml of 35-S-Met/Cys (Expre35-S35-S labeling mix, Perkin Elmer), 10% FBS, and Met/Cys-free DMEM was used for each sample. After radioactive pulse, each plate was washed thrice with PBS and chased with complete DMEM with an additional 20 mM Met/Cys for indicated times.

Cell lysates were prepared as described above and Cx32-Myc/His was isolated with Co2 + −agarose resin. Co2+ purified Cx32 was analyzed by western blotting. 35-S-labeled Cx32 was detected by exposing the blots overnight to a storage phosphor and then imaging with a Storm Phosphorimager (GE Life Sciences). Then the blots were probed with antibodies against the Myc tag as a loading control. 35-S-Cx32 and Cx32-Myc/His bands were quantified using image-J and normalized; that is, 35-S-Cx32 values were obtained for each sample by dividing 35-S-Cx32 band intensity by Cx32-Myc/His band intensity.

### Cell surface biotinylation

Biotinylation experiments were carried out 48 h post-transfection, following a 3 h treatment with TubA or DMSO. Confluent 60 mm plates of transfected cells were used for each experimental sample. Cell surface proteins were labeled with EZ-Link Sulfo-NHS-SS-Biotin (0.5 mg/ml; Thermo Scientific) for 30 min on ice. After biotinylation, cells were rinsed and blocked with PBS supplemented with 100 mM Glycine. Samples were lysed immediately or chased for 1.5 h with complete medium in the presence of TubA or DMSO, as indicated.

Cells were harvested and lysed in PBS supplemented with 1% Triton-X 100 and protease inhibitors, as described for immunoprecipitation. Aliquots of total protein were obtained prior to isolation of biotinylated proteins. Biotinylated proteins were isolated by binding to neutravidin-agarose beads (Thermo Scientific) and then eluted with SDS-PAGE sample buffer and subjected to western blotting along with total protein samples. Total protein samples were diluted accordingly to permit detection of both the eluate and total fractions using the same blotting conditions. Biotinylated and total Cx32 was detected by probing with an antibody against Myc-tag.

### Electrophysiology

Conductance measurements were made by pulsing from Vj = 0 to +/− 40 mV. After recording from cell pairs Cytoplasmic bridges were excluded by demonstrating the sensitivity of the recorded junctional conductance to application of bath solution containing octanol. Values for different pairing configurations were compared using the Kruskal-Wallis test with Dunn’s multiple comparison post-test. For determination of steady-state junctional conductance-junctional voltage (Gj-Vj) relations, cells were held between each pulse at Vj = 0 for 22.5 s, and cell 1 was pulsed in 20 mV increments from − 100 to + 100 mV for 12.5 s. Any resulting change in current in cell 2, the apposed cell, is attributable to junctional current. Currents are displayed with positive up for both cells; thus, a positive Vj step in cell 1 causes a downward deflection in cell 2. For WT and mutants with measurable conductance at Vj = 0 mV, voltage pulses were preceded by a short (~ 200 mS) pulse to − 20 mV for normalization. Data were collected as described. Steady-state (t = ∞) conductances were determined by fitting each current trace to a sum of exponentials. Dividing the current at t = ∞ by the applied voltage gives the steady-state junctional conductance.

For determination of Boltzmann parameters, steady-state plots were fit to the product of two Boltzmann distributions of the form:

$$ \mathrm{Gss}\left(\mathrm{V}\right)=\left\{\mathrm{Gmin}\left(+\right)+\left(\mathrm{Gmax}\left(+\right)-\mathrm{Gmin}\left(+\right)\right)/\right(1+\mathrm{e}\left[\mathrm{A}\left(+\right)\ \left(\mathrm{V}-\mathrm{V}0\left(+\right)\right)\right]\Big\}\ast \left\{\mathrm{Gmin}\left(-\right)-\mathrm{Gmin}\left(-\right)\Big)/\left(1+\mathrm{e}\left[\mathrm{A}\left(-\right)\left(\mathrm{V}-\mathrm{V}0\left(-\right)\right)\right]\right)\right\} $$

where Gss is the steady-state junctional conductance normalized to Vj = 0, Gmax is the maximal normalized conductance, and Gmin is the normalized residual conductance, which in macroscopic recordings is approached as the absolute value of Vj is increased, V_0_ is the voltage at which the conductance is 1/2 of the difference between Gmin and Gmax and roughly corresponds to the voltage at which a single connexin hemichannel has an open probability of 50%, and A is a parameter which reflects the slope of the Gj-Vj plot and is a measure of voltage sensitivity. A = nq/kT where n is the effective gating charge and q, k and T have their usual meanings. The (+) and (−) designations indicate that the parameters are fitted to the positive or negative limbs of the Gj-Vj relation. The model makes two assumptions that may not always be met for connexin gating: 1.The gating process has two states, 1where the energy difference between the states is proportional to the applied voltage, and 2: The steady state Gj-Vj arises from the gating of two independent apposed hemichannels. Whether or not assumptions 1 and 2 are met, the parameters generated provide a useful basis for comparison among channels produced by various pairing configurations of mutant and WT connexins. Homotypic pairs of Cx32WT- IRES-EGFP served as a positive control and pairs of empty vector expressing cells served as negative controls for homotypic pairing of mutants. For heterotypic cell pair controls, an additional “heterotypic” pairing of Cx32WT-IRES-EGFP with Cx32WT-IRES- DSRed expressing cells was performed. Pipette solution: 145 mM CsCl, 5 mM EGTA, 1.4 mM CaCl2, 5.0 mM HEPES pH 7.2; Bath solution: 150 mM NaCl, 4 mM KCl, 1 mM MgCl2, 2 mM CaCl2, 5 mM Dextrose, 2 mM Pyruvate, 5 mM HEPES; pH 7.4.

## Results

### HDAC6 inhibition causes Cx32 protein accumulation

Post-translational acetylation can regulate protein turnover [50–53]. Histone deacetylases (HDACs) form a functional class of cellular enzymes that remove acetyl groups from modified lysines of histones and other proteins. We chose inhibitors of HDACs as tools to investigate whether Cx32 is post-translationally acetylated and if this alters levels or stability of accumulated Cx32. Treatment of Cx32 transfected HeLa cells with a broad spectrum HDAC inhibitor, Trichostatin A (TSA), resulted in a 2.5-fold accumulation of Cx32 protein relative to untreated transfected cells (Fig. 1a). Histone Deacetylase 6 (HDAC6) is a cytoplasmic protein [54, 55] with several known *non*-histone cytoplasmic substrates [56–58]. Unlike other HDACs inhibited by TSA, HDAC6 inhibition does not alter histone acetylation. We therefore hypothesized that specific inhibition of HDAC6 would also result in the accumulation of acetylated Cx32 protein, independent of transcriptional perturbations that can result from broad spectrum TSA HDAC inhibition.

Treatment of transfected cells with the HDAC6-specific inhibitor, TubastatinA (TubA; Fig. 1b) increased the relative levels of Cx32 protein levels (3.7-fold±0.35 SEM, *N* = 5 independent experiments) compared to vehicle treated cells. To determine whether HDAC6 inhibition altered the half-life of Cx32, we performed 35-S pulse-chase experiments, comparing turnover of Cx32 in transfected cells treated with TubA to those treated with vehicle alone. 35-S labeled Cx32 detected by phosphorimaging was compared to total Cx32; Fig. 1c shows that half-life was increased by TubA treatment compared to untreated samples. To control for increased levels of Cx32 following TubA treatment, blots were normalized to total Cx32 for each treatment condition and reported as the percentage 35-S Cx32 remaining at *t* = 1.5 h. HDAC6 inhibition approximately doubled the amount of 35-S-Cx32 remaining at 1.5 h (from 40% in vehicle treated controls to 79% in TubA treated samples; Fig. 1d.). These results suggest that increased accumulation of Cx32 in response to HDAC6 inhibition results from stabilization of Cx32 protein rather than increased Cx32 transcription.

To confirm TubA activity, relative levels of Cx32 acetylation were determined using anti-acetylated lysine antibody (AcK) to probe western blots of immunoprecipitated Cx32. This revealed increased acetylation of Cx32 in concert with increases Cx32 protein in response to TubA inhibition of HDAC6 (Fig. 1e).

We next asked whether stabilization of Cx32 in Cx32-transfected cells resulted from reduced turnover of cell-surface-localized Cx32 or increased presence in other subcellular compartments. Cell-surface biotinylation experiments were carried out to determine if acetylated Cx32 was accumulating preferentially at the cell surface. This was assessed by comparing the cell surface-labeled Cx32 in cells that had either been treated with TubA or DMSO immediately after labeling and after a1.5 h chase period.

**Fig. 2:**
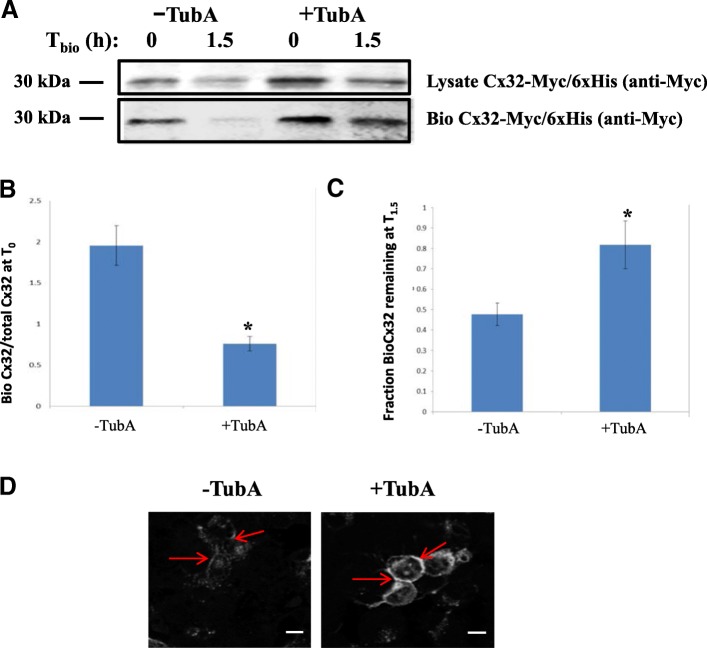
HDAC6 inhibition results in the accumulation of both cytoplasmic and cell surface Cx32. **a** TubA treatment stabilizes Cx32 levels as determined by increases in levels of biotinylated Cx32 at 1.5 h after labeling. N2a cell were transfected with Cx32-Myc/6xHis and treated with TubA or vehicle for 3 h prior to biotinylation of cell surface proteins. Following cell surface biotinylation, cultures were harvested and processed for total lysate (top) and biotinylated Cx32-Myc/6xHis (bottom) by Western blot, as described in Methods. **b** Densitometric quantification of Western blots shows a significant reduction in biotinylated Cx32/total Cx32 at T0 in TubA-treated samples as compared to vehicle-treated cells. **c** A significant increase in biotinylated Cx32/total Cx32 remained at T1.5 compared to vehicle-treated (*N* = 3 independent experiments, Student’s T-test *p < 0.05). **d** Immunostaining and confocal microscopy cell surface labeling of Cx32 confirmed TubA treatment increases Cx32 accumulation in cell surface and cytoplasmic compartments (see also Additional file 5: Fig. S1). Images shown were obtained at 63X magnification and scale bars = 25 μm. Arrows indicate points of cell-cell contact

Cx32-transfected cells were pre-treated with TubA or DMSO for three hours and subsequently labeled with Sulfo-NHS-SS-Biotin, a membrane impermeant biotin derivative, to tag cell surface proteins. Following cell-surface biotinylation, the cells were either lysed immediately (T_0_) or incubated an additional 1.5 h (T_1.5_) in the presence of TubA- or DMSO-containing medium to assess the total amount of biotinylated Cx32 from the cell surface labeled pool remaining at T_1.5_ following quenching. Biotinylated and total Cx32 were determined following western blot. Biotinylated Cx32 levels were normalized to total immunoprecipitated Cx32-Myc and expressed as the ratio biotinylated:total Cx32 (Fig. 2a). Vehicle-treated controls showed a higher ratio of biotinylated Cx32 to total Cx32 (2.0 ± 0.24 SEM, *N* = 3 independent experiments) at T_0,_ compared to TubA-treated cells (0.76 ± 0.09 SEM, *N* = 3 independent experiments) (Fig. 2b). Treatment with TubA resulted in elevated levels of biotinylated-Cx32 relative to total Cx32 at 1.5 h after cell-surface labeling; suggesting that inhibition of HDAC6 which increases relative levels of acetylated Cx32 also stabilizes the membrane-associated (biotinylated) pool of Cx32. At 1.5 h post-biotinylation, 82% (±12% SEM, *N* = 3 independent experiments) of biotinylated Cx32 relative to total Cx32 remained in TubA samples while only 48% (±5.5% SEM, *N* = 3 independent experiments) of biotinylated Cx32 remained in vehicle-treated samples (Fig. 2c); further supporting the conclusion that Cx32 levels are stabilized or enhanced by TubA inhibition of HDAC6 and that reduced acetylated-Cx32 at baseline in the absence of TubA (Fig. 1e) leads to loss of biotinylated Cx32 following quenching. Immunofluorescent staining and confocal imaging confirmed these results (Fig. 2d, Additional file 1: Figure S2, Additional file 2: Figure S3, Additional file 3: Figure S4 and Additional file 4: Figure S5). Cx32 staining in both cytoplasmic and cell surface pools was visibly increased after 6 h of TubA treatment (Fig. 2d, Additional file 1: Figure S2, Additional file 2: Figure S3, Additional file 3: Figure S4 and Additional file 4: Figure S5). Taken together, these results suggest that inhibition of HDAC6 decreases degradation and stabilizes Cx32, leading to its accumulation in both cell surface and cytoplasmic cell compartments.

### C-terminal lysines are implicated in HDAC6 inhibitor response

**Fig. 3:**
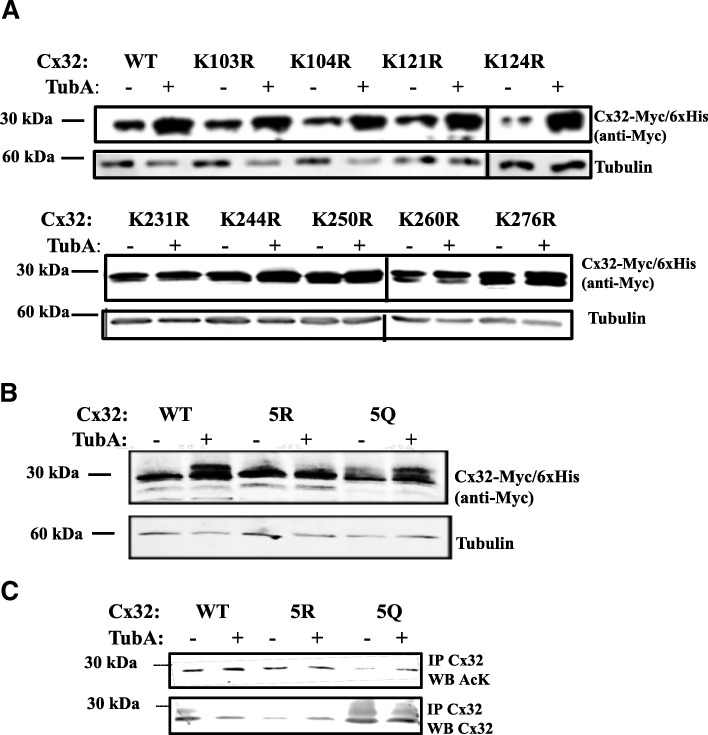
C-terminal lysines are required for HDAC6 inhibitor-induced accumulation of Cx32. **a** Mutation of C-terminal (bottom), but not cytoplasmic loop (top), lysines prevented Cx32 accumulation in response to TubA treatment. N2a cells transfected with either Cx32-Myc/6xHis or site-specific Cx32-Myc/6xHis K➔R acetylation-deficient mutants were treated with DMSO or TubA and processed for Western blot. Blots were probed for Myc-tag to detect Cx32, with beta-tubulin as a loading control. (Note that vertical lines between samples in figure indicate use of sister blots, run simultaneously.) (**b**) N2a cells were transfected with either Cx32-Myc/6xHis (WT), acetylation-deficient multi-site lysine to arginine mutant of all five cytoplasmic C-terminal lysines (5R), or acetylation-mimetic multi-site lysine to glutamine mutant of the five C-terminal lysines (5Q). WT, 5R, 5Q expressing cultures were treated with TubA or vehicle and assessed for Cx32 expression as in (**a**). **c** WT, 5R, 5Q expressing cultures were treated with TubA or vehicle and assessed for acetylation with anti-AcK. Cultures were immunoprecipitated with affinity purified rabbit-anti-Cx32 (E. Hertzberg) and probed with mouse monoclonal anti-Cx32 (7C6.7C; E. Hertzberg) or anti-AcK. Basal levels of acetylation were lower, as predicted, in untreated 5R and increased in 5Q relative to total Cx32. Treatment with TubA increased WT and 5Q acetylation, while 5R levels remained largely unchanged

Connexins contain three cytoplasmic domains (N-terminus, cytoplasmic loop, and C-terminus) that provide potential sites for regulation of protein turnover by post-translational modification. Nine lysines were identified in the cytoplasmic domains of Cx32. To determine if these residues were targets for acetylation and enhanced accumulation of Cx32, point mutations for each cytoplasmic lysine were generated and tested for resistance to the effects of HDAC6 inhibition. Mutation of lysine to arginine (K➔R) conserves the positive charge from lysine, but blocks acetylation [50, 59]. By contrast, lysine to glutamine mutations (K➔Q) are often mimetic of acetylation [59]. K➔R mutants in the cytoplasmic loop showed no differences compared to WT at baseline or following TubA (Fig. 3a, top), indicating the acetylation state of residues in this domain were not involved in mediating levels of Cx32 protein. Individual K➔R mutation of C-terminal lysines, however, reduced the TubA-stimulated accumulation of Cx32 protein, as compared to WT Cx32 (Fig. 3a bottom, 3B). To determine whether alteration of all five C-terminal lysines would further impact Cx32 accumulation, constructs in which all five C-terminal lysines were converted to arginine (5R) or glutamine (5Q) were tested for TubA induced accumulation of Cx32. Figure 3b shows that 5R Cx32 is resistant to TubA treatment, whereas HDAC6 inhibition in 5Q Cx32 results in increased levels of Cx32.

To determine whether modification of several C-terminal lysines was necessary for the effect, constructs in which all five C-terminal lysines were converted to arginine (5R) or glutamine (5Q) and probed for changes in acetylation. The double K231 + K260➔R mutant failed to detectably alter levels of Cx32 acetylation (Additional file 5: Figure S1), supporting the modification of all five C-terminal lysines. Cx32 was immunoprecipitated from transfected cells and acetylated Cx32 was detected by western blotting. We found that 5R Cx32 was still acetylated, but that this acetylation did not increase in response to TubA treatment (0.86 ± 0.15 SEM, 5R vs WT; *N* = 4 independent experiments) (Fig. 3c). This suggests that the observed increase in Cx32 acetylation and accumulation of Cx32 protein in response to HDAC6 inhibition was dependent upon C-terminal lysines. The relative acetylation level of 5Q Cx32 was 3.86-fold lower than WT (0.26 ± 0.15 SEM, 5Q vs WT; *N* = 4 independent experiments) but increased in response to HDAC6 inhibition (1.0 ± 0.28 SEM, 5Q vs WT; *N* = 4 independent experiments) (Fig. 3c). This is agreement with our finding that accumulation of 5Q Cx32 protein still occurred in response to HDAC6 inhibition (Fig. 3b). These results suggest that a subset of the C-terminal lysines could be acetylation sites, but other potential acetylation sites are available with HDAC6 inhibition under these experimental conditions.

To determine whether 5R and 5Q modification of C-terminal lysines altered acetylation, cells transfected with 5R and 5Q constructs were treated with TubA and probed for changes in Cx32 acetylation compared to WT. The relative acetylation level of the 5Q Cx32 acetylation mimetic was 3.86-fold lower than WT (0.26 ± 0.15 SEM, 5Q vs WT; *N* = 4 independent experiments) but increased in response to HDAC6 inhibition (1.0 ± 0.28 SEM, 5Q vs WT; *N* = 4 independent experiments) (Fig. 3c) in agreement with our finding that accumulation of 5Q Cx32 protein occurred in response to HDAC6 inhibition (Fig. 3b). The 5R Cx32 block mutant, however, was also acetylated, but this acetylation did not respond to HDAC6 inhibition with TubA (0.86 ± 0.15 SEM, 5R vs WT; *N* = 4 independent experiments) (Fig. 3c); suggesting that, under the experimental conditions described, other acetylation sites may become available with inhibition of HDAC6. Taken together, these results support the involvement of the C-terminal residues in the observed increase in Cx32 acetylation and accumulation of Cx32 protein following HDAC6 inhibition.

### C-terminal lysines regulate Cx32 ubiquitination and turnover

**Fig. 4:**
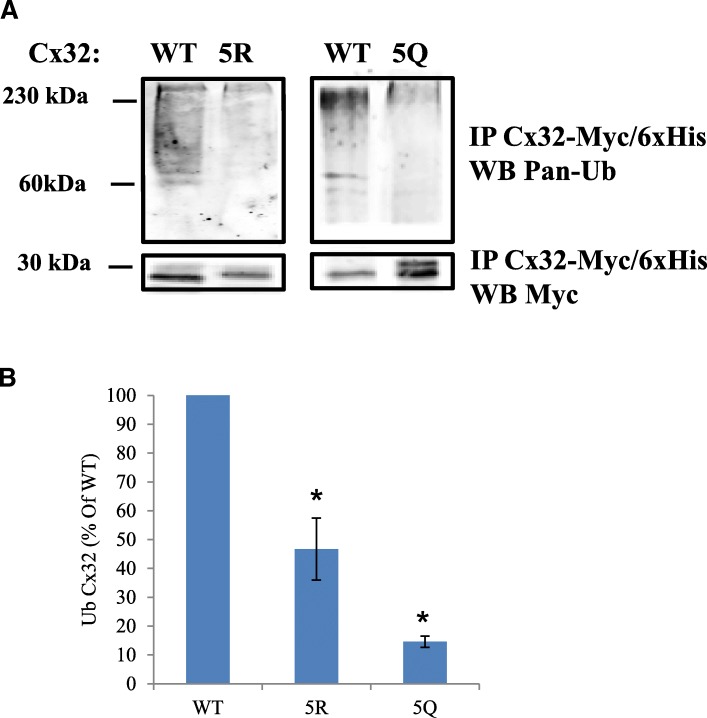
C-terminal lysines modulate Cx32 ubiquitination. **a** Ubiquitination levels of 5R, and 5Q Cx32, relative to WT, was assessed by probing Western blots of Co2+ affinity-purified Cx32-Myc/6xHis with antibodies against pan-ubiquitin and Myc-tag. Note that monoubiquitin has a molecular mass of 8.5 kDa and thus the bands shown here could represent polyubiquitinated and multi-mono-ubiquitinated Cx32. **b** Densitometric quantification of Ub-Cx32 as a percentage of WT Ub-Cx32 is shown here. Relative levels of Ub-Cx32 were 47% and 15% for 5R and 5Q, respectively, versus WT (*N* = 3 experiments, Student’s T-test: * *p* < 0.05)

**Fig. 5:**
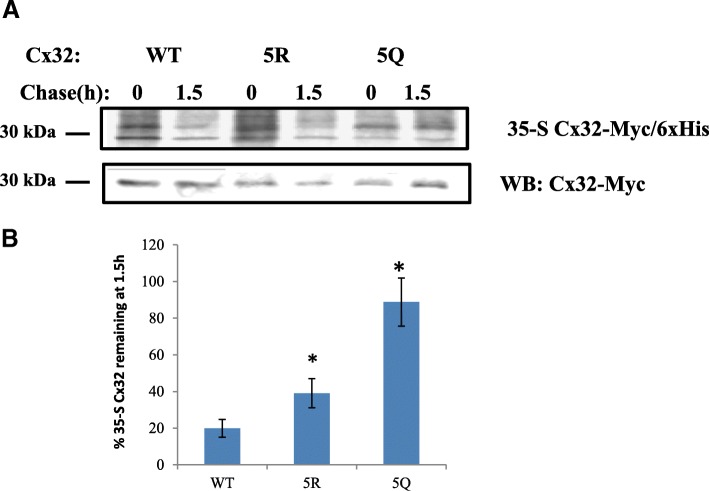
C-terminal lysines modulate Cx32 turnover. **a** Cells expressing Myc/6xHis tagged WTCx32, 5RCx32, or 5QCx32 were pulsed with 35-S-Met/Cys for 5 min and lysed (0) or chased 1.5 h in complete medium (1.5). Cx32-Myc/His was isolated by affinity purification and processed for Western blotting. 35S-Met/Cys incorporation into newly synthesized Cx32-Myc/His was detected by phosphorimaging and the blots were subsequently probed with anti-Myc antibody to detect Cx32-Myc/His. **b** Densitometric quantification of 35-S Cx32 remaining after 1.5 h. 35-S was quantified and normalized to total Cx32-Myc amount (*p < 0.05, Student’s T-test, *N* = 3)

Ubiquitination of lysines impacts protein turnover and can be regulated by lysine acetylation [60]. We hypothesized that hyper-acetylation of putative acetylation sites could reduce the availability of Cx32 ubiquitination sites, thereby reducing turnover of the protein. Levels of ubiquitination in the 5Q Cx32 mimetic was significantly lower relative to WT (15% +/− 2% SEM, *N* = 3 independent experiments) (Figs. 4a, b). Ubiquitination of 5R Cx32 block was reduced to 47% (+/− 15% SEM, *N* = 3 independent experiments) of the WT level, consistent with observed baseline acetylation levels (Fig. 4a, b). Given that a poly-ubiquitinated and multiple mono-ubiquitinated protein present similarly by SDS PAGE and Western blot, the possibility of a reduction in poly-ubiquination of 5R must be considered along with decreases in mono-ubiquination. 35-S pulse-chase experiments showed that replacement of C-terminal lysines with glutamine (5Q), in order to mimic constitutive acetylation, slowed down Cx32 by turnover to an even greater extent than the 5R acetylation block mutation (Fig. 5a, b.), despite the reduction in turnover with 5R compared to WT (Fig. 5a, b). Taken together, these results confirm our initial observations with HDAC6 inhibition of WT Cx32 and provide further support for that modulation of C-terminal lysines, either by acetylation or ubiquitination, modulates Cx32 protein.

### C-terminal lysines are dispensable for GJ function

**Fig. 6:**
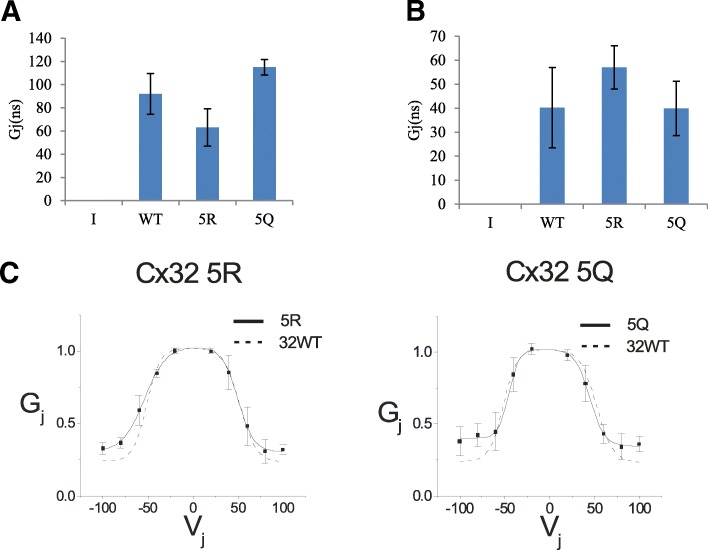
C-terminal lysine mutants form functional gap junctions. **a** Dual whole-cell recordings showed no significant differences in junctional conductance (Gj) between WT, 5R, or 5Q cell pairs. Mean Junctional conductances for pIRES2EGFP (I), WT, 5R, and 5Q Cx32 are plotted. **b** Cells in experiments plotted here were transfected with 3-fold more DNA than in A; no influence on Gj activity is observed. **c** Representative average normalized Gj-Vj relations with 5R (left) and 5Q (right) are compared with Cx32WT channels. The average normalized instantaneous (open triangles) and steady-state (filled squares) junctional conductance (Gj) at each Vj were calculated from current traces and fit as described in the Materials and Methods. Solid lines represent fits to Cx32-5R or Cx32-5Q; broken lines represent fits to data for Cx32WT homotypic cell-cell channels

To determine if the 5R and 5Q mutants altered formation of functional Cx32 GJ, we performed dual whole cell recordings to assess trans-junctional conductance and voltage gating. Dual whole cell recordings of trans-junctional conductance indicated that 5R and 5Q Cx32 formed functional GJ (Fig. 6a, b). Both Cx32 mutants generated trans-junctional voltages that were comparable to WT Cx32, indicating that cells expressing all three constructs were well coupled. We also evaluated the relationship between applied voltage and trans-junctional conductance to determine if the voltage gating of the either mutant differed from that of WT Cx32. The voltage gating of 5R and 5Q resembled WT Cx32, indicating that the mutations did not alter the structure of cytoplasmic motifs involved in voltage gating (Fig. 6c). This provided further evidence that neither the C-terminal lysines of Cx32, nor their post-translational modification, are required for normal GJ function. This is not surprising considering that the truncation of Cx32 at R220, has only a small effect on the steady state conductance- voltage relation.

### Cx32 acetylation regulates cellular proliferation

**Fig. 7:**
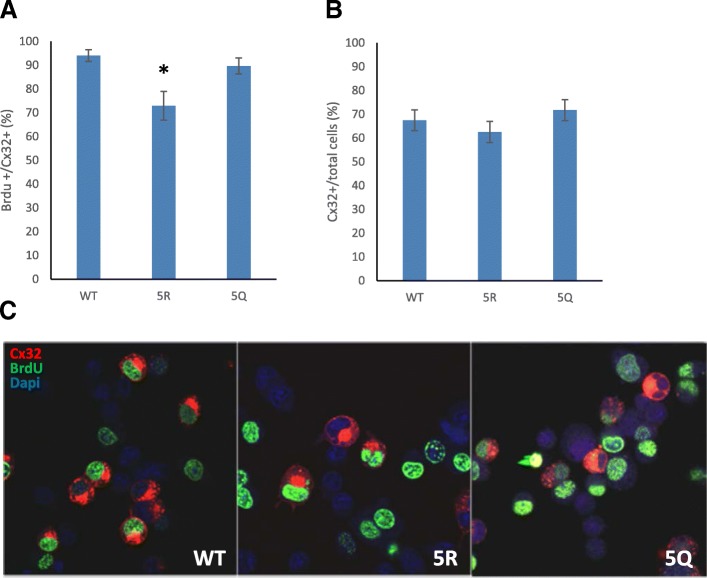
Cellular proliferation is regulated by Cx32 acetylation. BrdU incorporation by N2a cells expressing the pCx32-IRESEGFP WT, 5R, or 5Q constructs were quantified by immunofluorescent staining for Cx32 and BrdU, as described in methods. Following overnight incubation with medium containing BrdU, cells were fixed, labeled and scored for Cx32 expression and BrdU incorporation. The percentage of BrdU-positive and Cx32 co-expressing cells were normalized to the total number of Cx32-expressing cells for each construct. **a** The percentage of BrdU positive cells in 5R samples were significantly reduced compared to WT (*p < 0.0026). **b** No differences in the percentage of Cx32 positive cells per total DAPI positive nuclei (cell number) were noted. For each construct, three to four fields were imaged and quantified for each replicate coverslip per experiment using the MultiWavelength Cell Scoring app in Metamorph, as described in the Methods. (*N* = 3 experiments for each construct). **c** Representative images of BrdU incorporation by N2a cells expressing the pCx32-IRESEGFP WT, 5R, or 5Q constructs, labeled and processed as described above

Cytoplasmic domains of connexins have been implicated in a variety of physiological processes, such as the regulation of cellular proliferation, independent of their role in cell-cell communication [44, 61–63]. We hypothesized that acetylation of Cx32 could impact its role as a regulator of cellular proliferation. BrdU incorporation assays compared the fraction of proliferating cells found in cultures expressing WT, 5R, and 5Q Cx32. Expression of 5R Cx32 significantly reduced BrdU incorporation and immunolabeling (Fig. 7a, c) compared to WT and 5Q Cx32. This result suggests that blockade of Cx32 C-terminal acetylation can act as a modulator of channel-independent cellular processes, including cellular proliferation. To determine whether expression of WT, 5R, or 5Q Cx32 interfered with N2a cell survival, the percentage of Cx32 expressing cells versus total cells in each group was determined. We detected no difference between the percentage of cells expressing either mutant versus WT (Fig. 7b, c), suggesting that the reduction in BrdU incorporation observed in the 5R group was likely due to decreased proliferation rate rather than to toxicity of the mutant protein.

## Discussion

The data presented here address the role of C-terminal lysines and their post-translational modifications as regulators of Cx32 function. Our results demonstrate that acetylation of C-terminal lysines modulates levels and turnover of Cx32 protein in the cell. Changes in acetylation of these C-terminal lysines, in turn, alters ubiquitination of Cx32, such that levels of ubiquitination respond to occupancy or modification of these identified acetylation sites. Acetylation reduced turnover and increased accumulation of Cx32 protein throughout the cell. The acetylation state of C-terminal lysines did not affect functional GJ channel activity.

We utilized deacetylase inhibitors to promote the accumulation of acetylated Cx32 and determined that HDAC6 a regulator of Cx32 acetylation. Specific inhibition of HDAC6 stabilized Cx32 protein, causing it to accumulate throughout the cell, while increasing its acetylation and decreasing its ubiquitination. These results recapitulated our initial results with the broad type I and II deacetylase inhibitor, TSA. Our analysis of cytoplasmic lysine mutants of Cx32 revealed that C-terminal lysines are involved in the Cx32 accumulation which results from HDAC6 inhibition. Previous studies have shown that C-terminal truncation mutations of Cx32 retain the capacity to form functional GJ, with a resultant level of cell-cell coupling that is inversely proportional to the number of amino acids that were truncated [64, 65]. Furthermore, much of the truncated Cx32 accumulates in the cytoplasm, implicating the C- terminus in the regulation of GJ assembly and/or disassembly [66]. We found that the C-terminal K➔R and K➔Q mutations did not prevent Cx32 from reaching the cell surface (Additional file 1: Figure S2 and Additional file 2: Figure S3) but may alter the levels of plasma membrane Cx32 maintained over time. The results presented provide further support for a model of GJ assembly that is regulated by the C-termini of connexins. Specifically, post-translational modifications of C-terminal lysines modulate the amount of Cx32 maintained at the cell surface. This cell surface pool of Cx32 may be incorporated into GJ or may exist as hemichannels, either of which could regulate downstream physiological processes.

Our interpretation of the single residue K➔R mutations shown in Fig. 3a. is limited by the possibility that modifications at a given residue result in alterations of accessibility to or post translational modifications of other residues, thereby enhancing or masking the effect of a given mutation on the accumulation of Cx32 in response to HDAC6 inhibition. Additionally, it is possible that HDAC6 inhibition causes Cx32 accumulation through mechanisms that are both directly dependent upon Cx32 acetylation and dependent upon the hyperacetylation of other HDAC6 substrates. However, since we have shown that mutations that mimic constitutive acetylation of C terminal lysines (K➔Q) recapitulate the impact of HDAC6 inhibition on Cx32 turnover, ubiquitination and plasma membrane localization, the accumulation of Cx32 that is observed after HDAC6 inhibition can be largely attributed to direct modulation of post-translational modifications of Cx32.

Expression levels of 5R Cx32 acetylation were roughly equivalent to WT but did not increase in response to HDAC6 inhibition. This suggests that the five C-terminal lysines are acetylated in response to HDAC6 inhibition, and that other acetylated Cx32 lysine targets remain in the full-length protein. A possible source of this residual acetylation in the 5R mutant may be the result of additional or spurious acetylation of other lysines when normal sites are unavailable. 5Q Cx32 acetylation is visibly decreased as compared to 5R and WT, suggesting that the K➔Q mutation specifically succeeds in eliminating acetylation sites. Total acetylation levels of 5Q Cx32 increase after HDAC6 inhibition, with no concomitant increase in Cx32 protein accumulation.

We detected Cx32 as a doublet by Western blotting when cells expressing WT or TubA sensitive mutants were treated with TubA or when putative acetylation sites were mutated to glutamine (5Q) to mimic constitutive acetylation. It is possible that the higher molecular mass band in these samples is an additional post translationally modified form of Cx32. A previous study identified S240 as a phosphorylation site and C280/283 as palmitoylation sites [45]. Given the proximity of those residues to the putative acetylation sites identified here, it is conceivable that those or other post translational modifications could be positively regulated by acetylation.

Acetylation of Cx32 also appears to alter proliferation of transfected N2a cells. Specifically, expression of 5R, which blocks acetylation and conserves the charge properties of the mutated lysines, reduced proliferation, while proliferation in cells expressing the acetylation mimetic, 5Q, did not differ significantly from those expressing WT. Expression of Cx32 can act as either a positive or a negative regulator of proliferation depending upon the cell type and physiological context [6]. Our results suggest that acetylation of Cx32 is a modulator of proliferation and that the loss of acetylation sites suppresses proliferation of N2a cells. Thus, the acetylation state of C-terminal lysines in Cx32 has a role in regulating proliferation, an essential cell function. This modulation of proliferation rate may be occurring through acetylation specific protein-protein interactions rather than the general presence of lysines at the positions that we analyzed. Simply substituting a lysine with an amino acid that could not be otherwise modified (Cx32 5Q versus Cx32 5R), for example by ubiquitination, was not sufficient to suppress the proliferation modulating function of Cx32. Additionally, stabilization of Cx32 alone was not sufficient to modulate cellular proliferation because both 5Q and 5R Cx32 were more stable than WT and accumulated in the cell, but only Cx32 5R suppressed proliferation.

A recent study has identified several di-leucine-like motifs in the C terminus of Cx32 that regulate its endocytosis [67]. Two of our putative acetylation sites, K250 and K260, fall within two of these dileucine like motifs. Mutations within these motifs (L251A + L252A and L263A + R264A) increase GJ surface area and slow down turnover of Cx32 from the cell surface, due to reduced endocytosis. It is possible that cross talk between post translational modifications such as acetylation and ubiquitination involve these regulatory motifs.

Connexins are paradigmatic of a large cohort of proteins that are regulated by interactions with binding partners, which cause a particular sequence to change from an intrinsically disordered state into a functional secondary structure [68, 69]. For example, Stauch, et al. [69] report that binding of calmodulin (CaM) to the Cx32 C-terminus orders and stabilizes it into an alpha-helix. Chemical gating of Cx32 GJ occurs in the presence of high concentrations of Ca2+, which may lead to CaM activation and structural change in the C-terminus of Cx32 [43, 70]. Inhibition of CaM binding or truncation of CaM binding sites resulted in cytoplasmic accumulation of Cx32, further highlighting the regulatory role of interactions between Cx32 C- terminus and binding partners [66]. Other physiologically significant roles, such as the regulation of GJ assembly [64], cellular proliferation [44] and CO_2_ mediated GJ gating [71] have also been identified for the C-terminus of Cx32. However, little is known about the molecular mechanisms that control these functions, potentially through fine-tuning interactions between Cx32 and its binding partners. The results presented here suggest that the presence or absence of an acetyl group or the charged lysine in the C- terminus of Cx32 could be a modulator of interactions with binding partners, leading to alterations in Cx32 protein stability and physiological function.

## Conclusions

We have identified acetylation sites in the five lysines in the C-terminus of Cx32. Mutational analysis to acetylation deficient- or mimetic-residues demonstrates that these lysines play a potential role in regulating Cx32 ubiquitination, stability, and turnover. Inhibition of HDAC6 leads to accumulation of acetylated Cx32 protein, providing further evidence for HDAC6 modulation of Cx32 acetylation levels. Further analysis of the C-terminal mutants demonstrated that these critical lysine residues do not alter Cx32-mediated gap junction conductances, rather the acetylation state of these C-terminal lysines appears to regulate Cx32 protein turnover and to impact channel-independent functions, including cellular proliferation. These results highlight the role of lysines in the C-terminal tail of Cx32 in the fine-tuning of Cx32 stability and channel-independent functions.

## Additional files

Additional file 1: **Figure S2.** C-terminal lysines influence Cx32 localization and HDACi response. **(A.)** Cell surface biotinylation was performed in order to confirm the presence of WT, 5R, and 5Q Cx32 at the cell surface. Biotinylated and total Cx32 were detected by probing Western blots with an antibody against the Myc-tag. **(B.** and **C.)** N2A cells were transfected with WT, 5R, or 5Q and treated with TubA or vehicle (C), as described in methods. The amount of Cx32 at points of cellcell contact was measured by quantifying the fluorescence intensity of Cx32 antibody staining 48 h after transfection. **(B.)** Confocal images of Cx32 immunostaining, scale bars are 25 μm and arrows indicate points of cell-cell contact.**(C.)** Anti-Cx32 fluorescence intensity at points of cellcell contact was measured. Average fluorescence intensities at cell-cell contacts for each set of images is plotted. (*n* = 15 cell pairs for each group; *p < 0.05 compared to WT -TubA, Student’s T-test). (PDF 147 kb)

Additional file 2: **Figure S3.** C-terminal lysines influence Cx32 localization and HDACi response. Additional representative images of WT Cx32 expressing N2a cells (+/- TubA) shown in **Figure S2.** (PDF 244 kb)

Additional file 3: **Figure S4.** C-terminal lysines influence Cx32 localization and HDACi response. Additional representative images of 5R Cx32 expressing N2a cells (+/- TubA) shown in **Figure S2.** (PDF 317 kb)

Additional file 4: **Figure S5.** C-terminal lysines influence Cx32 localization and HDACi response. Additional representative images of 5Q Cx32 expressing N2a cells (+/- TubA) shown in **Figure S2.** (PDF 269 kb)

Additional file 5: **Figure S1.** Mutation of K231 and K260 does not eliminate acetylation. N2a cells were transfected with pIRESeGFP-Cx32 WT or K231+260R for 48 hours as described in methods section, then treated overnight with 20 μM Tubastatin. Cx32 was immunoprecipitated and blotted with indicated antibodies. (PDF 9 kb)

## Abbreviations

BrdU: Bromodeoxyuridine
Cx32: Connexin32
HDAC6: Histone deacetylase 6
N2a: Neuro2-A
TSA: TrichostatinA
TubA: Tubstatin A
