# Supplementary material for: Acetylation of C-terminal lysines modulates protein turnover and stability of Connexin-32
Source: BMC Cell Biol. 2018 Sep 29;19:22. doi: 10.1186/s12860-018-0173-0 (PMC6162937; doi:10.1186/s12860-018-0173-0)
Supplement: Supplementary file 1 — Figure S2. C-terminal lysines influence Cx32 localization and HDACi response. (A.) Cell surface biotinylation was performed in order to confirm the presence of WT, 5R, and 5Q Cx32 at the cell surface. Biotinylated and total Cx32 were detected by probing Western blots with an antibody against the Myc-tag. (B. and C.) N2A cells were transfected with WT, 5R, or 5Q and treated with TubA or vehicle (C), as described in methods. The amount of Cx32 at points of cellcell contact was measured by quantifying the fluorescence intensity of Cx32 antibody staining 48 h after transfection. (B.) Confocal images of Cx32 immunostaining, scale bars are 25 μm and arrows indicate points of cell-cell contact.(C.) Anti-Cx32 fluorescence intensity at points of cellcell contact was measured. Average fluorescence intensities at cell-cell contacts for each set of images is plotted. (n = 15 cell pairs for each group; *p < 0.05 compared to WT -TubA, Student’s T-test). (PDF 147 kb) [file 12860_2018_173_MOESM1_ESM.pdf]

Figure S2.

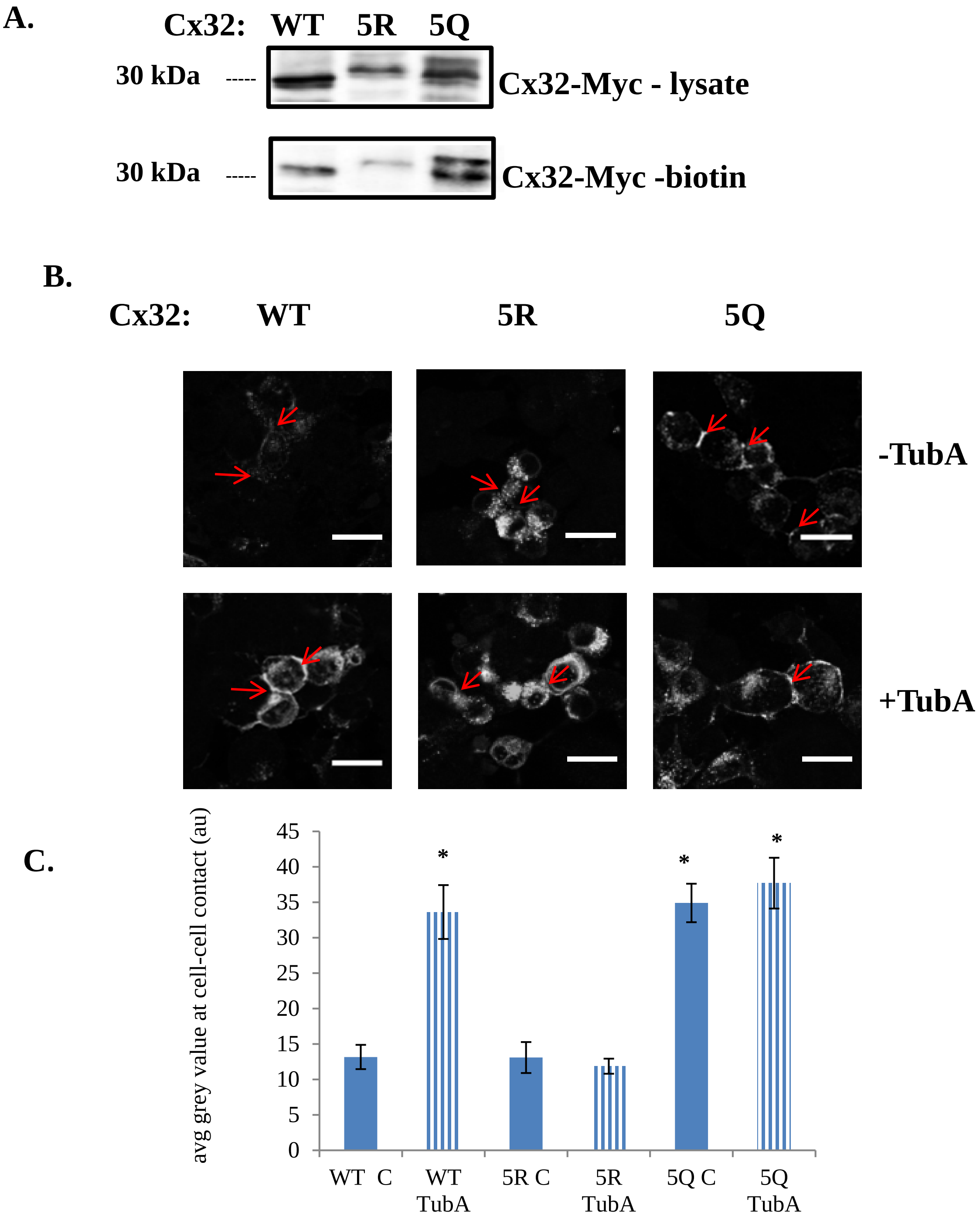

Figure S2. C-terminal lysines influence Cx32 localization and HDACi response. (A.) Cell surface biotinylation was performed in order to confirm the presence of WT, 5R, and 5Q Cx32 at the cell surface. Biotinylated and total Cx32 were detected by probing Western blots with an antibody against the Myc-tag. **(B. and C.)** N2A cells were transfected with WT, 5R, or 5Q and treated with TubA or vehicle (C), as described in methods. The amount of Cx32 at points of cell-cell contact was measured by quantifying the fluorescence intensity of Cx32 antibody staining 48h after transfection. **(B.)** Confocal images of Cx32 immunostaining, scale bars are 25µm and arrows indicate points of cell-cell contact.**(C.)** Anti-Cx32 fluorescence intensity at points of cell-cell contact was measured. Average fluorescence intensities at cell-cell contacts for each set of images is plotted. (n=15 cell pairs for each group; *$p<0.05$ compared to WT -TubA, Student's T-test) .
