# Supplementary material for: Acetylation of C-terminal lysines modulates protein turnover and stability of Connexin-32
Source: BMC Cell Biol. 2018 Sep 29;19:22. doi: 10.1186/s12860-018-0173-0 (PMC6162937; doi:10.1186/s12860-018-0173-0)
Supplement: Supplementary file 4 — Figure S5. C-terminal lysines influence Cx32 localization and HDACi response. Additional representative images of 5Q Cx32 expressing N2a cells (+/- TubA) shown in Figure S2. (PDF 269 kb) [file 12860_2018_173_MOESM4_ESM.pdf]

Figure S5.

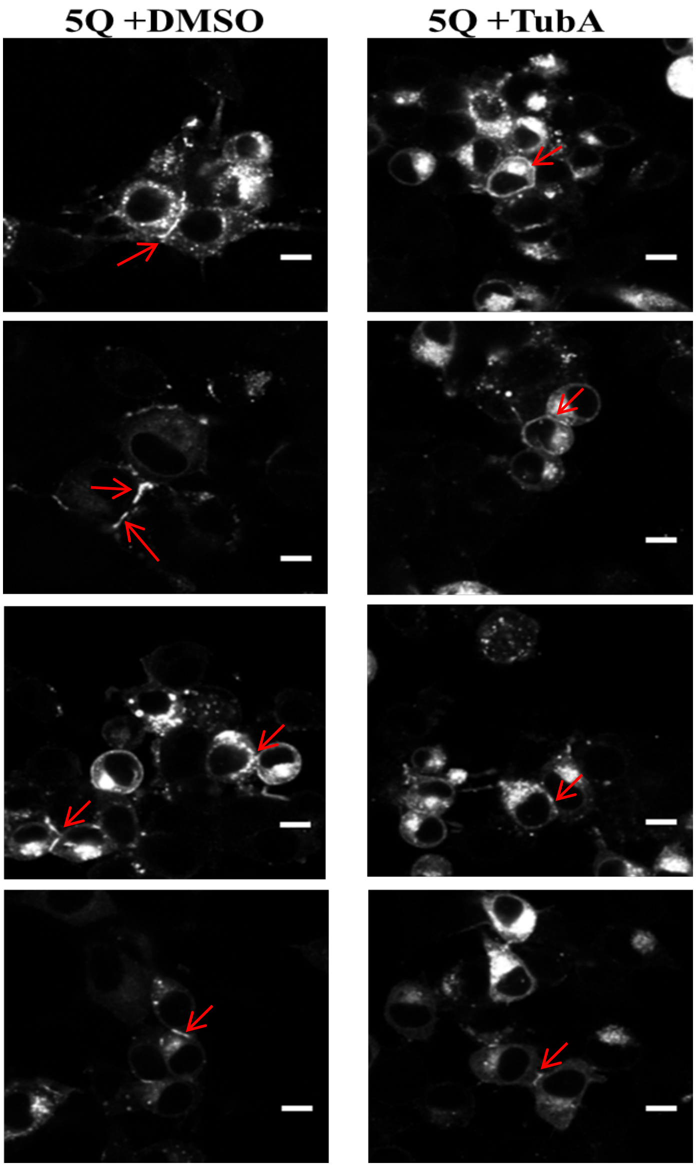

Figure S5. C-terminal lysines influence Cx32 localization and HDACi response. Additional representative images of 5Q Cx32 expressing N2a cells (+/- TubA) shown in Figure S2.
