# Supplementary material for: Acetylation of C-terminal lysines modulates protein turnover and stability of Connexin-32
Source: BMC Cell Biol. 2018 Sep 29;19:22. doi: 10.1186/s12860-018-0173-0 (PMC6162937; doi:10.1186/s12860-018-0173-0)
Supplement: Supplementary file 5 — Figure S1. Mutation of K231 and K260 does not eliminate acetylation. N2a cells were transfected with pIRESeGFP-Cx32 WT or K231+260R for 48 hours as described in methods section, then treated overnight with 20 μM Tubastatin. Cx32 was immunoprecipitated and blotted with indicated antibodies. (PDF 9 kb) [file 12860_2018_173_MOESM5_ESM.pdf]

Figure S1.

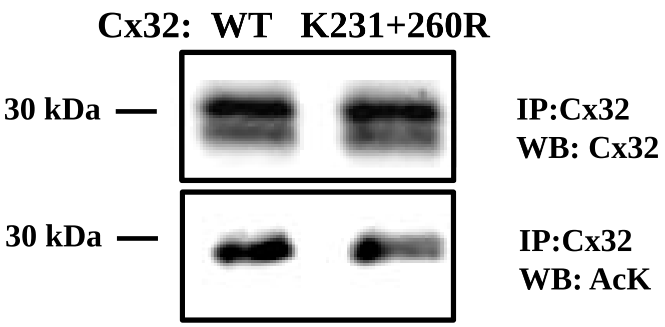

Figure S1. Mutation of K231 and K260 does not eliminate acetylation. N2a cells were transfected with pIRESeGFP-Cx32 WT or K231+260R for 48 hours as described in methods section, then treated overnight with 20 µM Tubastatin. Cx32 was immunoprecipitated and blotted with indicated antibodies.
